# Therapeutic Notes

**Published:** 1896-05-02

**Authors:** 


### THERAPEUTIC NOTES.

### PICRIC ACID IN THE TREATMENT OF BURNS.

The value of picric acid as an application for burns has more than once been noted in this journal. In the *Gazette des Hopitaux* of January 18th of this year, Thiery occupies nearly the entire space with an account of the picric acid dressing for burns of all degrees. He altogether repudiates the idea that picric acid is toxic or likely to explode. He has applied dressings of this acid to an area of the human body equal to one-third of the whole without any evil effects or indication of toxic symptoms. And with regard to the question of explosibility he draws attention to the distinction between picric acid and its salts, the former is perfectly free from danger. He contends that this acid has a keratoplastic action which is peculiar to itself and to one or two other bodies, namely, pyrogallic acid, ichthyol, and thyol, and to this property is due the rapid regeneration of epithelium cells when burns are treated by these substances. Further, picric acid has analgesic and antiseptic properties, which, in combination with its keratoplastic powers, are the three most important requirements of an application for a burn. The most painful of all burns are those which involve the nerve endings in the deeper layers of the skin, and in burns of this nature picric acid dressings are particularly valuable. The actual technique in the employment of the medium is fully described by Thiery. When possible, he recommends the immersion of the damaged part in a picric acid bath; the solution should be a saturated one, and the immersion should last from five to six minutes. Subsequently, if the integument is entire, the part should be enveloped in absorbent wool, otherwise in antiseptic gauze and an outside covering of wool. If it is impossible to make use of the bath, a compress of the same saturated solution should be applied and protected by a layer of oil silk and a coating of wool. The dressing should be renewed every third day. If a burn has been previously treated by other methods before applying the picric acid, care must be taken to see that all ointment or oil is removed. Another precaution that the author advises is to avoid touching healthy parts of the skin or clothing with the application, as a dark yellow stain is left wherever the acid touches.

